# Comparison of treatment outcomes in neck pain patients depending on the sex of the chiropractor: a prospective outcome study

**DOI:** 10.1186/s12998-017-0149-8

**Published:** 2017-07-17

**Authors:** Janine Thöni, Cynthia K. Peterson, B. Kim Humphreys

**Affiliations:** Department of Chiropractic Medicine, Orthopaedic University Hospital Balgrist, University of Zürich, Forchstrasse 340, 8008 Zürich, Switzerland

**Keywords:** Neck pain mechanical, Treatment outcome, Chiropractic, Spinal manipulative therapy, Gender

## Abstract

**Background:**

The high percentage of female chiropractic students in Switzerland suggests a future sex shift in the chiropractic profession in Switzerland. Thus the purpose of this study is to determine if male and female chiropractors achieve the same treatment outcomes in neck pain patients.

**Methods:**

Included in this prospective outcomes study were 849 patients with neck pain of any duration. Prior to the first treatment, baseline demographic data, the Bournemouth Questionnaire (BQ) and the numerical rating scale (NRS) for neck and arm pain were completed. At the follow-up time points of 1 week, 1, 3, 6 and 12 months, the Patient’s Global Impression of Change (PGIC) scale to categorize the actual ‘improvement’ and the BQ and the NRS for neck pain were completed.

The Chi-square test compared the proportion of patients reporting ‘improvement’ between male and female chiropractors for each time point. The unpaired Student’s t-test compared the BQ and the NRS actual and change scores between patients of male and female chiropractors at all time points. Demographic factors were compared between the sexes using the Chi-square test.

**Results:**

Proportionally more patients of female chiropractors reported ‘improvement’ at 1 month (*p* = 0.035) and significantly more pain reduction at 3 months (*p* = 0.040). Patients of male chiropractors presented with significant older age (*p* = 0.0001), higher levels of baseline neck pain (*p* = 0.012), a lower proportion with radiculopathy (*p* = 0.014) and less pain medication use (*p* = 0.046).

**Conclusions:**

Female chiropractors achieve at least equally satisfying treatment results for neck pain patients compared to male chiropractors. Female chiropractors also have a higher proportion of female patients compared to male chiropractors and patients presenting with radiculopathy and using pain medications.

**Trial registration:**

Not applicable for this type of study.

## Background

From the Swiss Job Analysis Survey 2009 it is known that there is a strong male predominance in the chiropractic profession in Switzerland [1]. This was also said to be found in the job analysis reports published for the United Kingdom and the United States, showing that there are more men than women working as chiropractors [2–4]. The sex ratio for the whole Swiss Association of Chiropractors in 2009 was 27% female versus 73% male chiropractors [1]. However, this will likely be changing in the future in Switzerland as currently, at the chiropractic program in the faculty of medicine at this major university, the majority of chiropractic students are female. Therefore a change in the sex distribution in the chiropractic profession in Switzerland is likely in the future. The data obtained in the Swiss Job Analysis study further shows some differences in practice characteristics between male and female chiropractors in Switzerland in terms of hours worked per week and number of patients treated per week. As the percentage of female chiropractors in Switzerland will rise in the future according to the sex ratio in the chiropractic education program, this could lead to a shortage of Swiss chiropractors in the future as women are more likely to work part time [1, 5–8]. In the general population, neck pain caused by musculoskeletal problems is extremely common [9, 10]. After low back pain, neck pain and its associated disability is the second most common chief complaint that leads to patients seeking chiropractic care [1, 11–13]. Previous research has already revealed predictors of positive outcome in neck pain patients, such as a short duration of neck pain as well as neck stiffness [14–16]. Reported prior improvement for both acute and chronic patients is also discovered to be a predictor of improvement in neck pain patients [16]. However, to date there has been no research on chiropractic treatment in neck pain patients investigating if the sex of the chiropractor is related to outcomes., This is relevant as at times there has been the misconception that at least some of the chiropractic treatments require a certain degree of strength to perform properly and that female chiropractors may find this more challenging compared to their male colleagues. Conversely, some patients comment that they prefer a female chiropractor as they assume her treatment may be gentler.

As the complaint of neck pain is common in the general population and as the percentage of female chiropractors is increasing in Switzerland, the hypothesis is that there is no difference in treatment outcomes in neck pain patients based on the sex of the chiropractor. Therefore, purpose of this study was to evaluate if the outcomes of neck pain patients treated by female chiropractors are the same as the outcomes of neck pain patients treated by male chiropractors.

## Methods

### Study design

This is a secondary analysis of data from a prospective cohort outcomes study [16] with follow-up time points of 1 week, 1 month, 3 months, 6 months and 1 year. Ethics approval for this study was obtained from the Canton of Zürich Switzerland ethics committee (EK-19/2009) and written informed consent was obtained from all patients.

### Patients

For this study, successive new patients were recruited from numerous chiropractic practices in Switzerland. The patients were over the age of 18, suffered from neck pain of any duration and had not received any chiropractic or manual therapy in the previous 3 months. Excluded were patients with specific pathologies of the cervical spine which are contraindications to chiropractic manipulative therapy. These included acute fractures, infections, tumours, inflammatory arthropathies, Paget’s disease, anti-coagulation therapy, cervical spondylotic myelopathy, known unstable congenital anomalies and severe osteoporosis [13].

All 260 members of the Association of Swiss Chiropractors were asked to recruit patients for this study and all examinations and treatments occurred in the participating chiropractic practices. All chiropractors received notification and instructions about this study plus the study protocol by email. Shortly before starting the data collection, details about the study were also discussed verbally at the annual mandatory post-graduate continuing education convention and any questions from the chiropractors were answered. At this information meeting as well as via email it was additionally emphasized that the participating chiropractors should not change their treatment methods, due to the fact that the purpose of this study was to evaluate outcomes as they were normally found in chiropractic practices. Therefore it was not desired to standardize neither the treatment method nor the treatment number. Nevertheless, it is known that most of the chiropractic patients in Switzerland (76–100%) are at least treated with the diversified technique [1].

### Baseline pain and disability data

Immediately before starting the treatment, every patient had to rate the severity of their current neck pain using the numerical pain rating scale (NRS) and a separate NRS for arm pain. The NRS is a scale evaluating the intensity of pain over the past 24 h, ranging from 0 (no pain) to 10 (worst pain imaginable).

Additionally, the Bournemouth Questionnaire for neck (BQN) disability, which has been validated and translated into German [17], was used for the baseline information. In each practice the questionnaires were distributed by the office staff. The BQN is a short-form multidimensional instrument and includes questions on pain, disability and also psychosocial topics. Altogether the BQN covers 7 domains by using an 11-point numerical rating scale (from 0 to 10) to evaluate each domain separately. These 7 domains include: (I) pain; (II) physical function (disability in activities of daily living (ADL)); (III) disability in social activities; (IV) anxiety; (V) depression; (VI) work-related fear avoidance (at home and at the workplace); and (VII) pain locus of control. In addition to the score obtained for each domain (with a maximum of 10 points each), the total score of all domains together (maximal 70 points) was also calculated. It has been shown, that the German version of the BQN is more responsive to change than the German versions of the Neck Pain and Disability (NPAD) questionnaire and the Neck Disability Index (NDI) for all scales [17]. Each of the 7 items of the BQN was designed to be used individually as well as the total score [18, 19].

### Clinical and demographic baseline data

At the first consultation, additional information was collected by the treating chiropractor and sent via fax to the university chiropractic research assistant. This supplementary data was completed in a baseline information form and included: patient age, sex, marital status, paid employment, whether or not the onset of pain was due to trauma, whether or not the patient smokes, whether or not the patient was currently taking pain medication, number of previous episodes, duration of current complaint, whether or not the patient had signs and symptoms of cervical radiculopathy, whether or not the patient complained of additional dizziness and the patient’s general state of health. This data was used in the original predictors of outcome paper [16]. Only patient age, sex, duration of complaint, medication use and the presence of radiculopathy were included in this secondary analysis of outcomes based on the sex of the treating chiropractor.

### Outcome measures

To evaluate how much the patients ‘improved’ overall compared to their condition prior to treatment at the various outcome time points, the Patient’s Global Impression of Change (PGIC)-scale was used [20, 21]. The PGIC-scale is a 7-point rating scale to report the patient’s improvement from just prior to the start of treatment to the current time point ranging from 1 (much better) to 7 (much worse). Scores of 1 (much better) or 2 (better) on the PGIC-scale were categorized as ‘improved’ (primary outcome), whereas scores of 3 (slightly better), 4 (no change), 5 (slightly worse), 6 (worse) and 7 (much worse) were defined as ‘not improved’ [17]. A score of 5, 6 or 7 was additionally categorized as worsening (secondary outcome). This scoring system has been used in other studies [16, 22].

Additional outcomes were assessed using the NRS (neck) and the BQN scores at the follow up time points and their subscale change (baseline minus 1 week, 1 month, 3 months, 6 months or 1 year) scores (secondary outcomes).

The outcome data collection (1 week, 1 month, 3 months, 6 months and 1 year) after the start of the treatment was done by research assistants at the university hospital who were unknown to the patients. Via telephone interviews to the patients, they collected the data of the NRS neck pain, NRS arm pain, Patient Global Impression of Change (PGIC) scale and the BQN. The patients knew that they were enrolled in the outcomes study, having signed informed consents. This occurred at the point of data collection for the original outcomes study, and therefore patients were not nformed that subsequent analysis would include comparing treatment outcomes between male and female chiropractors.

### Statistical analysis

Baseline factors with categorical variables were compared between patients of male and female chiropractors using the Chi-square test. For continuous variables (i.e. change scores/normally distributed data) the unpaired Student’s t-test was used. To analyze differences within patient groups for continuous variables, the paired t-test was performed. The proportion of patients reporting ‘improvement’ or ‘worsening’ was compared between the sexes of the chiropractors for each data collection time point using the Chi-square test. The NRS and BQ actual and change scores were compared between the patients of male and female chiropractors at each time point using the unpaired Student’s t-test. SPSS version 21.0 (IBM, Armonk, New York) was used for the statistical analysis.

## Results

### Baseline characteristics

Of the 260 active members of the Association of Swiss Chiropractors, 78 contributed patients to this study (30% response rate), with 46 chiropractors being male and 32 being female. As a total of 70 of the 260 Swiss chiropractors were female and 190 were male, this corresponds to a 45.7% response rate for the female chiropractors and a 24.2% response rate for the male chiropractors. Baseline data was provided for 849 neck pain patients with 450 being treated by male and 399 being treated by female chiropractors.

Overall there were more women (65%, *N* = 551) than men (35%) suffering from neck pain in this study. Additionally, there was a statistically significant difference in the proportion of male versus female patients treated when comparing the sexes of the chiropractors. Female chiropractors treated significantly more female (71.2%) than male (28.8%) neck pain patients compared to male chiropractors (59.3% female and 40.7% male patients) (*p* = 0.0001). At baseline, neck pain patients of male chiropractors were significantly older than patients treated by female chiropractors (*p* = 0.0001) (Table 1) and they reported statistically significantly higher levels of neck pain compared to the patients of their female co-workers (*p* = 0.012) (Table 1). All scores on the Bournemouth Questionnaire at baseline were compared between the two groups, but there were no significant differences.

**Table 1:** Baseline Characteristics

	Male DCs	Female DCs	*p*-value
Patient sex	40.7% male (*n* = 183)	28.8% male (*n* = 115)	0.0001*
	59.3% female (*n* = 267)	71.2% female (*n* = 284)	
Patient age (y), (SD)	43.15 (SD 13.659)	39.71 (SD 13.637)	0.0001*
Radiculopathy present	11.2% (*n* = 49)	17.3% (*n* = 69)	0.014*
Pain medication use	27% (*n* = 121)	35% (*n* = 140)	0.046*
Chronicity <4 weeks (acute)	44.9% (*n* = 197)	44.1% (*n* = 174)	0.02*
Chronicity 4–8 weeks (subacute)	8.9% (*n* = 39)	12.9% (*n* = 51)	
Chronicity 8–12 weeks (subacute)	11.4% (*n* = 50)	6.1% (*n* = 24)	
Chronicity >12 weeks (chronic)	34.9% (*n* = 153)	37.0% (*n* = 146)	
Baseline NRS (SD)	5.951 (SD 2.211)	5.561 (SD 2.256)	0.012*
BQ1 (pain)	5.680 (SD 2.262)	5.651 (SD 2.306)	0.853
BQ2 (disability in ADL)	4.185 (SD 2.823)	3.999 (SD 2.764)	0.337
BQ3 (disability in social activities)	3.614 (SD 3.120)	3.433 (SD 3.079)	0.400
BQ4 (anxiety)	5.382 (SD 2.883)	5.344 (SD 2.827)	0.851
BQ5 (depression)	3.441 (SD 3.099)	3.376 (SD 3.066)	0.762
BQ6 (work realted fear avoidance)	4.442 (SD 2.936)	4.577 (SD 3.064)	0.515
BQ7 (pain locus of control})	5.035 (SD 2.864)	4.916 (SD 2.846)	0.548
Baseline BQ total (SD)	31.761 (SD 15.124)	31.319 (SD 15.396)	0.676

*p ≤ 0.05, n = number of patients, *SD* standard deviation, *y* year, *NRS* numerical rating scale, *BQ* Bournemouth Question, *DC* Doctor of Chiropractic. Italic and bold = *p* < 0.05

There was also an association between patients diagnosed with radiculopathy and consulting a female chiropractor. A higher proportion of patients seeing female chiropractors presented with the clinical signs and symptoms of radiculopathy (17.3%) compared to the patients of male chiropractors (11.2%) (*p* = 0.014) (Table 1).

Comparing the duration of symptoms of the two groups showed that the percentage of acute and chronic patients was distributed fairly equally between male and female chiropractors. A significant difference in chronicity was only found in the two subgroups of the subacute category, with male chiropractors having more patients suffering from neck pain at 8–12 weeks, but having less patients complaining of neck pain at 4–8 weeks (*p* = 0.02) (Table 1).

### Differences in patients reporting ‘improvement’ or ‘worsening’ treated by male or female chiropractors

A significant difference in the percentage of patients reporting clinically relevant ‘improvement’ was discovered at 1 month, where the patients of the female chiropractors did significantly better (*p* = 0.035) (Table 2). At 1 week, 3 months, 6 months and 1 year, there were no significant differences in the outcomes between the two groups (Table 2).

**Table 2:** Comparison of proportion of patients ‘improved’, ‘not improved’ and ‘worse’ based on PGIC responses

	PGIC	Male DC’s	Female DC’s	*p*-value
At 1 week	improved	56.5%	54.6%	0.863
	not improved	43.5%	45.4%	
	worse	4.3%	4.2%	1.000
At 1 month	improved	69.2%	76.7%	0.035*
	not improved	30.8%	23.3%	
	worse	3.8%	1.8%	0.161
At 3 months	improved	74.9%	81.4%	0.053
	not improved	25.1%	18.6%	
	worse	4.8%	1.2%	0.012*
At 6 months	improved	78.0%	78.6%	0.840
	not improved	22.0%	21.4%	
	worse	2.9%	2.2%	0.710
At 1 year	improved	81.3%	80.6%	0.957
	Not improved	18.7%	19.4%	
	worse	3.3%	2.7%	1.000

*p ≤ 0.05, *PGIC* Patient’s Global Impression of Change, *DC* Doctor of Chiropractic. Italic and bold = *p* < 0.05

Comparing reported ‘worsening’ between the two groups revealed a significant difference in worsening at 3 months. A higher proportion of patients treated by male chiropractors (4.8%) reported worsening compared to patients treated by female chiropractors (1.2%). At all the other time points, no significant difference in worsening between the two groups could be detected (Table 2).

### Secondary outcome differences

One month after the start of treatment, patients treated by male chiropractors reported significantly higher scores on the BQN for pain, disability in activities of daily living, social disability, pain locus of control, BQN total score as well as in the NRS for neck pain compared to the patients of female chiropractors (Table 3). The same results were found at the follow-up time point of 3 months (Table 3). At 6 months, the BQN total score was almost significant (*p* = 0.051) (Table 3).

**Table 3:** Comparing NRS- and BQN-Scores at 1 week, 1 month, 3 months, 6 months and 1 year between male and female Chiropractors

	Patients of Male DCs mean (SD)	Patients of Female DCs mean (SD)	*p*-value
At 1 week NRS neck score	3.628 (SD 2.265)	3.407 (SD 2.335)	0.222
At 1 week BQ1 (pain)	4.529 (SD 2.160)	4.517 (SD 2.252)	0.943
At 1 week BQ2 (disability in ADL)	2.983 (SD 2.629)	2.978 (SD 2.740)	0.984
At 1 week BQ3 (disability in social activities)	2.298 (SD 2.857)	2.472 (SD 3.047)	0.455
At 1 week BQ4 (anxiety)	4.052 (SD 2.701)	3.924 (SD 2.799)	0.554
At 1 week BQ5 (depression)	2.081 (SD 2.638)	2.175 (SD 2.796)	0.657
At 1 week BQ6 (work-related fear avoidance)	3.529 (SD 2.874)	3.709 (SD 3.035)	0.446
At 1 week BQ7 (pain locus of control)	4.571 (SD 3.023)	4.518 (SD 3.081)	0.828
At 1 week BQ total score	24.060 (SD 13.172)	24.357 (SD 14.309)	0.783
At 1 month NRS neck score	2.879 (SD 2.249)	2.442 (SD 2.146)	0.008*
At 1 month BQ1 (pain)	3.043 (SD 2.175)	2.720 (SD 2.135)	0.046*
At 1 month BQ2 (disability in ADL)	1.787 (SD 2.375)	1.330 (SD 2.135)	0.007*
At 1 month BQ3 (disability in social activities)	1.348 (SD 2.384)	1.003 (SD 2.131)	0.044*
At 1 month BQ4 (anxiety)	2.572 (SD 2.736)	2.240 (SD 2.579)	0.099
At 1 month BQ5 (depression)	1.401 (SD 2.352)	1.156 (SD 2.206)	0.156
At 1 month BQ6 (work-related fear avoidance)	2.630 (SD 2.741)	2.301 (SD 2.745)	0.115
At 1 month BQ7 (pain locus of control)	3.516 (SD 3.197)	3.033 (SD 3.263)	0.048*
At 1 month BQ total score	16.324 (SD 13.742)	13.765 (SD 13.148)	0.012*
At 3 months NRS neck score	2.402 (SD 2.308)	1.866 (SD 2.084)	0.002*
At 3 months BQ1 (pain)	2.562 (SD 2.403)	2.016 (SD 2.105)	0.002*
At 3 months BQ2 (disability in ADL)	1.225 (SD 2.024)	0.891 (SD 1.771)	0.022*
At 3 months BQ3 (disability in social activities)	0.762 (SD 1.781)	0.483 (SD 1.486)	0.027*
At 3 months BQ4 (anxiety)	2.005 (SD 2.515)	1.660 (SD 2.370)	0.066
At 3 months BQ5 (depression)	1.066 (SD 2.092)	0.869 (SD 1.886)	0.199
At 3 months BQ6 (work-related fear avoidance)	1.858 (SD 2.535)	1.964 (SD 2.694)	0.601
At 3 months BQ7 (pain locus of control)	2.787 (SD 3.043)	2.266 (SD 2.789)	0.021*
At 3 months BQ total score	12.274 (SD 12.527)	10.119 (SD 11.531)	0.020*
At 6 months NRS neck score	2.191 (SD 2.235)	1.949 (SD 2.155)	0.151
At 6 months BQ1 (pain)	2.292 (SD 2.307)	2.067 (SD 2.271)	0.199
At 6 months BQ2 (disability in ADL)	1.081 (SD 1.965)	0.857 (SD 1.713)	0.115
At 6 months BQ3 (disability in social activities)	0.700 (SD 1.742)	0.478 (SD 1.542)	0.080
At 6 months BQ4 (anxiety)	1.949 (SD 2.491)	1.639 (SD 2.352)	0.097
At 6 months BQ5 (depression)	1.119 (SD 2.183)	0.860 (SD 1.913)	0.103
At 6 months BQ6 (work-related fear avoidance)	2.045 (SD 2.735)	1.747 (SD 2.532)	0.147
At 6 months BQ7 (pain locus of control)	2.511 (SD 2.956)	2.197 (SD 2.832)	0.162
At 6 months BQ total score	11.675 (SD 12.739)	9.822 (SD 11.956)	0.051
At 1 year NRS neck score	2.155 (SD 2.278)	1.833 (SD 2.182)	0.066
At 1 year BQ1 (pain)	2.283 (SD 2.341)	2.051 (SD 2.281)	0.200
At 1 year BQ2 (disability in ADL)	0.978 (SD 1.882)	0.831 (SD 1.870)	0.316
At 1 year BQ3 (disability in social activities)	0.595 (SD 1.621)	0.524 (SD 1.691)	0.581
At 1 year BQ4 (anxiety)	2.267 (SD 2.782)	1.902 (SD 2.595)	0.085
At 1 year BQ5 (depression)	1.057 (SD 2.105)	0.892 (SD 2.016)	0.309
At 1 year BQ6 (work-related fear avoidance)	1.892 (SD 2.652)	1.763 (SD 2.609)	0.534
At 1 year BQ7 (pain locus of control)	2.231 (SD 2.825)	2.017 (SD 2.709)	0.330
At 1 year BQ total score	11.322 (SD 12.542)	9.967 (SD 12.503)	0.167

*p ≤ 0.05, *SD* standard deviation, *NRS* numerical rating scale, *BQ* Bournemouth Question, *DC* Doctor of Chiropractic, *ADL* activities of daily living. Italic and bold = *p* < 0.05

The change scores were used to assess how much the BQN and NRS neck scores changed from baseline to the various outcome time points between the two groups. Only the change scores of the time points in which there was a significant difference between the two groups in the NRS and BQN scores above were assessed (1 month and 3 months). By 1 month after the start of treatment, no significant difference was found in the change scores between the two groups treated by male or female chiropractors, although the BQ6 evaluating the change in work-related fear avoidance was almost significant (*p* = 0.052) (Table 4). At 3 months, the change score for BQ1 (pain) was significantly higher within patients of female chiropractors, indicating a higher amount of neck pain reduction.

**Table 4:** Comparing NRS- and BQ-change-scores from baseline to 1 and 3 months between male and female chiropractors

	Patients of male DCs mean (SD)	Patients of female DCs mean (SD)	*p*-value
1 month NRS neck change score	3.079 (SD 2.936)	3.077 (SD 2.636)	0.993
1 month BQ1 change score (pain)	2.593 (SD 2.867)	2.898 (SD 2.699)	0.147
1 month BQ2 change score (disability in ADL)	2.262 (SD 3.249)	2.621 (SD 2.803)	0.118
1 month BQ3 change score (disability in social activities)	2.140 (SD 3.446)	2.493 (SD 3.138)	0.158
1 month BQ4 change score (anxiety)	2.721 (SD 3.351)	3.003 (SD 3.199)	0.256
1 month BQ5 change score (depression)	1.954 (SD 2.924)	2.139 (SD 2.926)	0.404
1 month BQ6 change score (work-related fear avoidance)	1.714 (SD 3.424)	2.214 (SD 3.336)	0.052
1 month BQ7 change score (pain locus of control)	1.536 (SD 3.878)	1.893 (SD 4.061)	0.237
1 month BQ total change score	14.912 (SD 17.481)	17.277 (SD 16.445)	0.065
3 month NRS neck change score	3.519 (SD 2.982)	3.614 (SD 2.787)	0.667
3 month BQ1 change score (pain)	3.102 (SD 3.094)	3.564 (SD 2.712)	0.040*
3 month BQ2 change score (disability in ADL)	2.893 (SD 3.336)	3.073 (SD 3.057)	0.463
3 month BQ3 change score (disability in social activities)	2.848 (SD 3.347)	3.011 (SD 3.187)	0.517
3 month BQ4 change score (anxiety)	3.367 (SD 3.472)	3.623 (SD 3.250)	0.322
3 month BQ5 change score (depression)	2.353 (SD 3.055)	2.458 (SD 3.016)	0.652
3 month BQ6 change score (work-related fear avoidance)	2.542 (SD 3.437)	2.475 (SD 3.485)	0.803
3 month BQ7 change score (pain locus of control)	2.290 (SD 3.894)	2.673 (SD 3.840)	0.201
3 month BQ total change score	19.415 (SD 17.930)	20.945 (SD 16.829)	0.252

*p ≤ 0.05, *SD* standard deviation, *NRS* numerical rating scale, *BQ* Bournemouth Question, *DC* Doctor of Chiropractic, *ADL* activities of daily living. Italic and bold = *p* < 0.05

## Discussion

As the purpose of this study was to compare treatment outcomes in Swiss neck pain patients treated by male versus female chiropractors, it was reassuring, both for patients and clinicians, to find that the neck pain patients treated by female chiropractors reported a significantly higher proportion of patients ‘improved’ only at the 1 month outcome time point compared to patients treated by their male colleagues. At the later data collection time points there were no significant differences in the percentage of patients reporting ‘improvement’ between male and female chiropractors, although the PGIC-result at 3 months was almost significant in favor of female chiropractors’ patients. However, there was a significant difference in the proportion of patients reporting ‘worsening’ at the 3 month time point with patients treated by male chiropractors being 4 times more likely to report ‘worsening’ (i.e. 4.8% of patients) compared to patients treated by female chiropractors (i.e. 1.2% of patients). Both of these values are very low however compared to the proportion of ‘improved’ patients for both groups of clinicians.

The same supporting results were found in the BQ- and NRS neck data where patients of female chiropractors had better scores at 1 month and also at 3 months. Contributing to the significant difference in outcome of the NRS neck data at 1 and 3 months in favor of female chiropractors may be the fact that the NRS neck pain score was significantly higher in male chiropractors’ patients at baseline and also continued to be significantly higher after 1 and 3 months of treatment. This suggests that the pain reduction within male and female chiropractor’s patients over time was most likely similar considering that patients of male chiropractors presented with higher levels of pain. Indeed, the fact that the NRS change scores at 1 and 3 months did not show any significant differences between patients of male and female chiropractors supports this conclusion.

As mentioned above, 4 subscales and the total score of the Bournemouth Questionnaire (pain, disability in ADL, disability in social activities, pain locus of control) also showed significantly lower scores in patients seen by female chiropractors compared to those seen by male chiropractors after 1 and 3 months. However, a look at the actual change scores of the BQ-data does not confirm significant differences between male and female chiropractors’ patients over time. Although statistically significant differences were found for several of the outcome measures between the patients of male and female chiropractors, when looking at the actual numerical differences, it could be argued that they are not clinically relevant. Other than the BQ total scores, the actual numerical differences were less than 0.6 of an NRS point. With large sample sizes such as those in this study, it is not difficult to find statistically significant differences. However, authors and readers must be cautious about implying too much clinical relevance in such cases when there is less than a 1 point difference on the NRS scale.

It has been suggested that the change in pain over time may be more relevant to the patient and gives a better clinical picture than the pain level at a single point in time [23]. This suggests that the change scores and the PGIC-results are more meaningful in this study than the BQ- and NRS actual neck results at the various time points. Therefore, the most important results are the significant ‘improvement’ after 1 month followed by the significant BQ1 change score after 3 months of treatment, both representing better findings within female chiropractors’ patients. Nevertheless, all scores 6 months and 1 year after the start of treatment were similar in patients seen by male and female chiropractors.

Patient management not only consists of good manual treatments, but the communication style plays a conceivably important role as well. Doctor-patient communication may be an additional and important factor in order to get a good and satisfying result at the end of a treatment period. It has been shown that female physicians view doctor-patient communication in a more positive way, that they talk more often and longer to patients and that they set a higher value on psychosocial aspects, confidence and empathy when talking to patients [24, 25]. This may be a reason why female chiropractors achieved slightly better results in their neck pain patients after 1 month. But as the satisfaction was very high not only within female, but also within male chiropractors’ patients at all other time points, good care seems to be provided by both genders.

The baseline data showed that consulting a chiropractor because of neck pain was more common in women than men (65% versus 35%). This result is supported by other studies which report that in general predominantly women suffer from neck pain [10, 23, 25, 26]. The fact that women tend to have smaller and weaker neck muscles compared to men, but not a comparatively smaller or lighter head is a plausible explanation for a higher amount of vulnerability of female necks and therefore neck pain found in the literature [27].

Additionally, a strong relationship between patient sex and chiropractor sex was revealed in this study. Although more females than males suffered from neck pain, female chiropractors still had a significantly higher percentage of female patients than male chiropractors. Previous studies already investigated if there is a correlation between patient and doctor sex and discovered a “gender concordance preference” in females [28–31]. One reason why women often tend to consult a female rather than a male physician is the opinion that doctors of the same gender are more empathic and easier to talk to. This makes the patient feel more comfortable and at ease during the case history and especially during the examination [28]. Particularly for health professions dealing with more intimate and psychosocial health problems, “gender concordance preference” seems to play a major role [32]. As chiropractic is a profession with a lot of physical contact, it is very likely that some women prefer female chiropractors for the above-mentioned reasons.

Some studies have indicated that not only in general but also with neck pain, women report significantly higher pain and disability levels than men [25, 27, 33]. On the other hand, a study by Peterson et al. showed no difference in pain levels between male and female neck pain sufferers [23] and in this current study the male patients presented with higher levels of pain. As there is little and inconclusive information about pain level differences in neck pain patients depending on their sex, it is not possible to identify if this has an effect on the outcome of this study.

Interestingly, the results showed that female chiropractors had significantly more patients diagnosed with radiculopathy. However, their outcomes were better in the short term. One low back pain study showed that chiropractic patients with low back pain and additional leg pain improved less than patients without leg pain [34], while other studies did not support this result [35, 36]. Additionally, Swiss neck pain patients with arm pain undergoing chiropractic treatment have been shown to improve as much as patients without arm pain [16]. This suggests that the significant difference in radiculopathy between patients of male and female chiropractors in this present study did not have an influence on the better outcomes of female chiropractors’ patients in the short term.

Whether or not neck pain is of a long or short duration has been shown to have an impact on the treatment outcome. While patients suffering from acute neck pain present with higher pain and disability levels, they improve faster and in a higher proportion than chronic neck pain sufferers, although many chronic neck pain patients improve as well [14, 16]. Thus a shorter duration of neck pain at baseline has been shown to result in a more favorable outcome [14]. This has also been found in cervical disc herniation patients undergoing chiropractic treatment in Switzerland [37]. However, the significance shown in the chronicity categories of this present study did not seem to have an impact on the outcome as the proportion of acute and chronic patients was the same for male and female chiropractors. Although the results showed a significant difference in the distribution of the acute, subacute and chronic patients between patients seen by male and female chiropractors, this significance is of minor importance as it only involved the two subacute classifications which had the least number of patients overall.

Pain medication use in this present study was more common in patients of female chiropractors at baseline. This is explained by the findings that female chiropractors are visited more commonly by female rather than male neck pain sufferers. As stated by several studies, women use significantly more prescription and non-prescription analgesics than men [38–40]. This may also have contributed to the differences in baseline pain levels reported in this current study where the male patients reported higher pain scores. Because of the correlation between women and use of analgesics reported in the mentioned studies it can be suggested that the higher percentage of female patients in female chiropractors’ practices is the reason why female chiropractors have more patients using analgesics. To date there are no studies investigating if the additional use of analgesics leads to a better outcome in chiropractic patients than chiropractic treatment alone. Thus it cannot be clarified if the higher amount of analgesics use in female chiropractors’ patients influences the outcome positively.

It is possible that the age of patients has an influence on the outcome with older patients improving less than younger patients. Female chiropractors have significantly younger patients than male chiropractors and it is possible that this would be related to the better outcome in patients of female chiropractors. However, the actual numeric age difference between patients seen by male and female chiropractors is small as the large sample size in this study may have made this significant. Thus, the age difference in this study also should not impact the outcome relevantly.

It was interesting to observe that there was a higher response rate from female chiropractors compared to males contributing patients to this study. This is likely due to the fact that female Swiss chiropractors are more likely to work part time compared to their male colleagues [1]. No information is available comparing characteristics between chiropractors who contributed patients to this study and those who did not.

### Limitations

As this study was not a randomized controlled trial but a prospective cohort study, no control or comparison group was included. Therefore, whether the improvement that occurred in both study groups is mainly a result of the chiropractic treatment or only a consequence of the natural history of healing cannot be verified. Chiropractors from all parts of Switzerland contributed patients to this study. These patients chose their own chiropractor so randomization was not possible in this setting. Therefore, it is likely that inequalities between the two study groups additionally influenced the results. By analyzing the unbalanced baseline data and their possible impact on the results in the discussion section, possible additional sources of influence were identified, but some may have remained undetected.

In this study, data collection at baseline was conducted with a paper questionnaire and the follow-up data was collected by telephone interviews. It has been shown that using telephone interviews for data collection has a slight positive effect on the results, as patients tend to report better scores when answering the questions by telephone than in a written form [41–43]. By using research assistants for the telephone interviews who were unknown to patients and chiropractic practices, an attempt was made to minimize this effect. Furthermore, anonymity was ensured.

As there was no standardization of the treatment method desired in this study and as the chiropractors did not have to describe their treatment procedures, it is unknown which treatment method was used by how many chiropractors and if one procedure achieved better results than others. Whether male and female chiropractors in this study used the same or different treatment methods could therefore also not be determined. Nevertheless, from the results of the Swiss Job Analysis 2009 it can be assumed that the vast majority of the patients in this study were treated with at least the diversified technique [1].

The participating chiropractors contributed different numbers of patients to this study, as the number of patients was not determined. Therefore, chiropractors contributing numerous patients to this study did have a higher impact on the overall outcome than chiropractors contributing only few patients to this study. Additionally, the number and frequency of treatments was not determined. This decision was left to the chiropractor, as the purpose of this study was to evaluate outcomes as they would be found routinely in chiropractic practice.

Thirty percent of chiropractors (78 of 260) in Switzerland contributed patients to this present study. Whether or not these 30% truly represented the average of Swiss chiropractors remains unknown.

## Conclusions

The results showed that the sex of the chiropractor was only linked to the treatment outcomes of these chiropractic neck pain patients at the 1 month time point. Additionally, although the BQ neck pain change score at 3 months also favored the female chiropractors, the sample size was very large in this study and the actual numerical differences were comparatively small, though statistically significant. Therefore these neck pain change scores at 3 months are not likely to be clinically relevant. Nevertheless, this study has shown that with their treatment of neck pain patients, female chiropractors achieve at least equally satisfying results as their male colleagues. This suggests that the quality of the chiropractic treatment of neck pain patients in Switzerland will not change in the future despite a prospective sex shift in the chiropractic profession.

## Abbreviations

ADL: Activities of daily living
BQ: Bournemouth Questionnaire
BQ1: Bournemouth Question 1
BQN: Bournemouth Questionnaire for Neck Pain
DC: Doctor of Chiropractic
NDI: Neck Disability Index
NPAD Scale: Neck Pain and Disability Scale
NRS: Numerical pain rating scale
PGIC Scale: Patient’s Global Impression of Change Scale
