# Correction to: Comparison of treatment outcomes in neck pain patients depending on the sex of the chiropractor: a prospective outcome study

**DOI:** 10.1186/s12998-017-0164-9

**Published:** 2017-11-13

**Authors:** Janine Thöni, Cynthia K. Peterson, B. Kim Humphreys

**Affiliations:** Department of Chiropractic Medicine, Orthopaedic University Hospital Balgrist, University of Zürich, Forchstrasse 340, 8008 Zürich, Switzerland

## Correction

After publication of the original article [1] the authors noted that the presentation of the name B. Kim Humphreys was incorrect.

The authors name should be presented in the original article as:

**First name:** B. Kim.

**Surname:** Humphreys.

This has been corrected in the authorship list and reference of this Correction.

## References

[1] Thöni J, Peterson CK, Humphreys BK (2017). Comparison of treatment outcomes in neck pain patients depending on the sex of the chiropractor: a prospective outcome study. Chiropr Man Ther.

